# Apolipoprotein E, low-density lipoprotein receptor, and immune cells control blood-brain barrier penetration by AAV-PHP.eB in mice

**DOI:** 10.7150/thno.46992

**Published:** 2021-01-01

**Authors:** Bao-Shu Xie, Xin Wang, Yao-Hua Pan, Gan Jiang, Jun-Feng Feng, Yong Lin

**Affiliations:** 1 Department of Neurological Surgery, Ren Ji Hospital, School of Medicine, Shanghai Jiao Tong University, 160 Pujian Road, Shanghai 200127, P.R. China.; 2 Department of Anesthesia, Ren Ji Hospital, School of Medicine, Shanghai Jiao Tong University, 160 Pujian Road, Shanghai 200127, P.R. China.; 3 Department of Pharmacology, School of Medicine, Shanghai Jiao Tong University, 280 South Chongqing Road, Shanghai 200025, P.R. China.; 4 Department of Neurological Surgery, First Affiliated Hospital of Sun Yat-Sen University, 58 Zhongshan 2nd Road, Guangzhou, Guangdong 510080, P.R. China.

**Keywords:** blood-brain barrier, AAV-PHP.eB, apolipoprotein E, low-density lipoprotein receptor, immune cells

## Abstract

**Rationale:** The blood-brain barrier (BBB) prevents the effective delivery of therapeutic molecules to the central nervous system (CNS). A recently generated adeno-associated virus (AAV)-based vector, AAV-PHP.eB, has been found to penetrate the BBB more efficiently than other vectors including AAV-PHP.B. However, little is known about the mechanisms. In this study, we investigated how AAV-PHP.eB penetrates the BBB in mice.

**Methods:** We injected AAV-PHP.eB into the bloodstream of wild-type C57BL/6 and BALB/c mice as well as mouse strains carrying genetic mutation in apolipoprotein E gene (*Apoe*) or low-density lipoprotein receptor gene (*Ldlr*), or lacking various components of the immune system. Then, we evaluated AAV-PHP.eB transduction to the brain and spinal cord in these mice.

**Results:** We found that the transduction to the CNS of intravenous AAV-PHP.eB was more efficient in C57BL/6 than BALB/c mice, and significantly reduced in *Apoe* or *Ldlr* knockout C57BL/6 mice compared to wild-type C57BL/6 mice. Moreover, poor CNS transduction in BALB/c mice was dramatically increased by B-cell or natural killer-cell depletion.

**Conclusions:** Our findings demonstrate that the ApoE-LDLR pathway underlies the CNS tropism of AAV-PHP.eB and that the immune system contributes to the strain specificity of AAV-PHP.eB.

## Introduction

The blood-brain barrier (BBB), a physical lining between the circulation system and the central nervous system (CNS), prevents many water-soluble molecules and even lipid-soluble molecules with large molecular weight from entering the CNS freely 1. Thus, researchers and clinicians have been eager to explore the vehicles that could deliver therapeutic molecules through the BBB effectively, so as to treat diseases of the CNS 2,3.

Potential vehicles including viral vectors and nanoparticles have been found or engineered, but their barrier-penetrating efficiency still falls short of expectations. For example, adeno-associated virus (AAV), a non-enveloped, single-strand DNA molecule 4.7kb long, has received much attention due to its non-pathogenicity, low immunogenicity, and long-lasting expression 4,5. AAV is commonly used in the lab for gene transfer and in the clinical trials of gene therapies. Twelve AAV serotypes, from AAV-1 to AAV-12, have been reported in humans and nonhuman primates 6,7. Each of them is characterized by a tropism for specific cell types. Among them, AAV-9 has proven able to penetrate the BBB and transduce the CNS cells. However, this BBB penetration occurs only at a very high virus load and with limited CNS transduction efficiency 8-10. One of the reasons for this low efficiency is that the systemically administered AAV can trigger the host immune response, which eliminates the vector before it can cross the BBB 11.

Using a technique called Cre recombination-based AAV targeted evolution (CREATE), a recent study created a new AAV variant, AAV-PHP.B, which is 40 times more efficient than AAV-9 at transferring genes to the CNS 12. Nevertheless, its BBB penetration is strain- and species-dependent, with great efficiency in C57BL/6 mice and poor efficiency in BALB/c mice and marmosets 13,14. A novel variant, AAV-PHP.eB, with amino acids DGT instead of AQT at positions 587-589 of the AAV-PHP.B capsid sequence, has since been identified and shown greater barrier penetration than AAV-PHP.B 15. While this finding is encouraging, understanding the mechanisms of this CNS tropism will make the researchers easier to improve the design of AAV-based gene delivery vectors to the CNS.

Several pathways allow certain molecules to cross the BBB, including paracellular aqueous pathway, transcellular lipophilic pathway, membrane transporters pathway, adsorptive transcytosis, and receptor-mediated transcytosis 16. Recent studies have reported that the tropism of AAV-PHP.B and AAV-PHP.eB for the CNS is driven by a cellular receptor, lymphocyte activation protein-6A (LY6A), on the BBB 17-19. In fact, in addition to LY6A, there are many other receptors, such as low-density lipoprotein receptor (LDLR), related to receptor-mediated transcytosis on the endothelial cells of the BBB 20,21. In addition, LDLR, with its ligand apolipoprotein E (ApoE), plays a role in virus cellular transduction 22. For instance, it has been shown that the entry of the hepatitis C virus (HCV) into hepatocytes is determined by the interaction of a lipo-viro-particle (LVP), formed by HCV and Apo proteins including ApoE, with LDLR and heparan sulfate proteoglycans (HSPGs) on the hepatocytes' surface 22.

Thus, in this study, we explored whether ApoE and its receptor, LDLR, played critical roles in the CNS transduction of intravenously administered AAV-PHP.eB. We also examined what role the host immune system may play in limiting the ability of this vector to transduce the CNS. To test these hypotheses, we injected AAV-PHP.eB vector into mouse strains carrying mutation in *Apoe* or *Ldlr* gene, or lacking various components of the immune system. We found that the CNS transduction efficiency of intravenous AAV-PHP.eB depended on the ApoE-LDLR pathway and was limited by natural killer (NK) cells and B cells, but not T cells. Our findings indicate that AAV-PHP.eB relies on the ApoE-LDLR pathway to transduce the CNS and suggest that host immunity may partly account for the strain specificity of the AAV-PHP.eB capsid.

## Materials and methods

### AAV Virions

The AAV-PHP.eB vector, pAAV-CMV-*mScarlet*-3FLAG, used in our present study was purchased from the OBIO, Shanghai, China. It contains a common and efficient cytomegalovirus (CMV) promoter, the gene for a bright red fluorescent protein (*Scarlet*), and three *flag* genes. This vector was co-transfected into 293T cells with a plasmid carrying AAV replication and capsid genes as well as with a helper plasmid. 3 days (d) later, the cells were collected to extract inactive virions, AAV-PHP.eB particles.

Virions based on an AAV-PHP.eB vector carrying the gene for the enhanced green fluorescent protein (*Egfp*) were prepared in the same fashion.

### Animals

Male mice weighing 20-30g were randomly chosen in this study. Wild-type C57BL/6, wild-type BALB/c, BALB/c nude, and C.B-17 SCID mice were obtained from Shanghai laboratory Animal Co., Ltd. (SLAC, Shanghai, China). Both *Apoe* and *Ldlr* knockout (*Apoe^-/-^* and *Ldlr^-/-^*) animals in a C57BL/6 genetic background were from Nanjing Biomedical Research Institute of Nanjing University (Nanjing, China). In addition, by breeding *Ldlr^-/-^* C57BL/6 and wild-type BALB/c mice, we created new heterozygous knockout (*Ldlr^-/+^*) mice carrying the *Ldlr* gene from the BALB/c background.

All animals were housed in a 12-hours (h)/12-h light-dark cycle, and the food and water were provided* ad libitum*. All procedures were approved by the Shanghai Jiao Tong University School of Medicine and were performed in accordance with the Guide for Care and Use of Laboratory Animals.

### Genotyping

Mouse genotypes were verified using 1cm long tail clips, which were lysed for DNA extraction using the Rapid Animal Genomic DNA Isolation Kit (Sangon Biotech, Shanghai, China), and subjected to polymerase chain reaction (PCR) on a Bio-Rad C1000 Touch™ Thermal Cycler (Bio-Rad Laboratories, Inc., Hercules, CA, USA). The following primers were used for the *Apoe^-/-^* and wild-type mice: forward: 5'-TGCCTAGTCTCGGCTCTGAACTAC-3'; reverse: 5'-CAACCTGGGCTACACACTAATTGAG-3'. Meanwhile, three primers were designed for the *Ldlr^-/-^* and wild-type mice: one common forward primer: 5'-TATGCATCCCCAGTCTTTGG -3', one wild type reverse primer: 5'-CTACCCAACCAGCCCCTTAC-3', and one mutant reverse primer 5'-ATAGATTCGCCCTTGTGTCC -3'.

PCR cycling conditions for the *Apoe* genes were: 1) 5 minutes (min) at 95 °C for initial activation step; 2) 30 seconds (s) at 95 °C for denaturation; 3) 30 s at 58 °C for annealing; 4) 45 s at 72 °C for extension; repeat steps 2-4 for 35 cycles; 5) A final extension at 72 °C for 5 min.

For *Ldlr* genes, touchdown PCR (TD-PCR) was performed: 1) Initial activation at 94 °C for 2 min; 2) Denaturation at 94 °C for 20 s; 3) Annealing for 15 s, starting temperature at 65 °C, followed by 0.5 °C decrease per cycle; 4) Annealing at 68 °C for 10 s; repeat steps 2-4) for 10 cycles; 5) Denaturation at 94 °C for 15 s; 6) Annealing at 60 °C for 15 s; 7) Extension at 72 °C for 10 s; repeat steps 5-7) for 28 cycles; 8) A final extension at 72 °C for 2 min.

PCR products were analyzed by electrophoresis in 1.5% agarose gels.

### Administration of AAV virions

AAV-PHP.eB or AAV-9, diluted in 0.9% NaCl to a final volume of 250 microliters (µL) and with a final titer of 3 × 10^11^ viral genomes (v.g.), was injected to each mouse via the tail vein using a 29-gauge needle.

### Evaluation for the BBB permeability

The permeability of the BBB was assessed by injecting Evans Blue (MilliporeSigma, St. Louis, MO, USA) intravenously. Briefly, 30 min after AAV-PHP.eB was given to a wild-type C57BL/6 mouse, the mouse was also injected with Evans Blue (2%, 4mL/kg) into its left internal jugular vein. 2 h later, the mouse was anesthetized with sodium pentobarbital (70 mg/kg, intraperitoneally) and transcardially perfused with phosphate-buffered saline (PBS). The brain was removed, weighed, and homogenized in 1 mL of 50% trichloroacetic acid buffer, followed by centrifugation at 15,000 × *g* for 30 min. The supernatant was recovered and diluted with ethanol at a ratio of 1 to 3. Evans Blue in the supernatant was quantified by measuring the absorbance of the solution at 610 nm in a spectrophotometer (BioTek, Winooski, VT, USA). Evans Blue amount was expressed by micrograms (µg) per gram of mouse brain.

### Plasma preparation and rescue experiments

1 milliliter (mL) of whole blood was drawn from the cardiac ventricle of wild-type C57BL/6, wild-type BALB/c and *Apoe^-/-^* mice into a sodium-heparin tube. It was then transferred into a clean 1.5 mL microfuge tube. After the tube was centrifuged at 2,000 × *g* for 15 min, approximately 300 µL of supernatant plasma sample was taken out and stored at 4 °C for later use.

For the rescue experiment, 125 µL of the plasma sample was first mixed with 125 µL of AAV-PHP.eB solution, producing a final viral titer of 3 × 10^11^ v.g., at room temperature (RT) for 30 min. The mixture was then injected into the tail veins of *Apoe^-/-^* mice.

### *Ldlr* gene sequencing

To find the potential difference between *Ldlr* gene sequences of C57BL/6 mice and those of BALB/c mice, gene sequencing was carried out. Total RNA was isolated from the brains of C57BL/6 and BALB/c mice with TRIzol reagent (Invitrogen, Thermo Fisher Scientific Inc., Carlsbad, CA, USA). After RNA samples were determined to be pure, double-strand cDNA was synthesized using the PrimeScript Double Strand cDNA Synthesis Kit (TaKaRa Bio Inc., Kusatsu, Shiga, Japan). For each mouse, the *Ldlr* gene was amplified by three PCR reactions, followed by DNA sequencing. Finally, the entire sequences of the *Ldlr* gene from the two mouse strains were analyzed with the Basic Local Alignment Search Tool (BLAST).

### Western blots for the *Ly6a* gene expression

After being anesthetized, wild-type C57BL/6, wild-type BALB/c, and *Apoe^-/-^* and *Ldlr^-/-^* mice were transcardially perfused with 0.9% NaCl. The mouse brains were removed and homogenized in RIPA lysis buffer (Beyotime, Jiangsu, China). The supernatant was collected, its protein concentration was measured with Pierce BCA protein assay kit (Thermo Fisher Scientific Inc., Waltham, MA, USA), and the LY6A level was evaluated by western blots. Briefly, 20 µg of protein samples were loaded and separated on a 12% SDS-polyacrylamide gel. After the proteins were transferred onto a PVDF membrane, the blot was blocked in 5% non-fat milk and then incubated with rabbit anti-LY6A primary antibody (Ab) (ab109211, abcam, Cambridge, MA, USA) overnight at 4 °C. After three washes, the blot was incubated with secondary antibody at RT for 2 h. LY6A was detected with Pierce™ ECL Western Blotting Substrate (Thermo Fisher Scientific Inc., Waltham, MA, USA). Tubulin was used as an internal control for each experiment and detected with rabbit anti-β3-Tubulin Ab (#5568, Cell Signaling Technology, Inc., Danvers, MA, USA) and HRP-conjugated goat anti-rabbit IgG H&L (ab205718, abcam, Cambridge, MA, USA).

The NIH ImageJ (1.52a) program was used for protein band densitometric analysis. The relative density of the LY6A band was determined using a ratio of the LY6A optical density to that of the β3-Tubulin in the same protein sample.

### Depletion of NK cells in BALB/c mice

NK cells were depleted in four BALB/c mice via intraperitoneal injection of 20 µL of anti-asialo GM1 Ab (Wako, Chuo-Ku, Osaka, Japan) in 250 µL of 0.9% NaCl. According to the product datasheet, this treatment depletes NK cells efficiently for 4 d. Thus, flow cytometry was used to verify the NK-cell depletion 4 d after the administration of the Ab. A dramatic reduction in the number of NK cells was noted in the spleen.

Another four BALB/c mice were then depleted of NK cells using the same procedure, and 4 d later given 3 × 10^11^ v.g. of AAV-PHP.eB via the tail veins. The same dose of the Ab was reinjected at d 5, 10, and 15 after the first Ab injection to maintain the NK-cell depletion. 3 weeks (w) post-AAV-PHP.eB injection, samples from both the blood and the spleen were collected to confirm the NK-cell depletion by flow cytometry, and the mouse brains were removed for sections.

### B-cell depletion in BALB/c mice

BALB/c mice were injected intraperitoneally with an anti-CD20 murine IgG2a monoclonal Ab, 5D2 (250 µg in 250 µL of 0.9% NaCl), a generous gift from Genentech Inc (Genentech, South San Francisco, CA, USA). The effect on B-cell depletion was examined by the flow cytometry at 3 d and 3 w after the single Ab injection. Significant depletion was observed by d 3 and persisted to w 3.

Another group of BALB/c mice was then given 3 × 10^11^ v.g. of AAV-PHP.eB 3 d post-Ab injection to evaluate the influence of B cells on the virus penetration through the BBB. BALB/c mice without B-cell depletion were used as control.

### Flow cytometry

Samples of the spleen and the blood were collected for the flow cytometry from anesthetized mice at 3 d post B-cell depletion, 24 d after B-cell depletion/ 21 d (3 w) after AAV-PHP.eB injection, and 4 d of the NK-cell depletion. While the blood sample was simply collected, the removed spleen was cut into pieces and ground. Then, the samples were passed through 70 microns (um) nylon mesh for single-cell suspensions, followed by centrifugation at 300 × *g* for 10 min. Red blood cells in the sample were lysed with cell lysis buffer (559759, BD Pharmingen, San Jose, CA, USA), and the remaining cells were washed three times and counted (1×10^6^ cells in a 100 µl for staining).

For B-cell staining, the antigens on the cell surface were labeled with anti-B220 (1 µg/100 µl, APC, BD Biosciences, San Jose, CA, USA), anti-CD3 (1 µg/100 µl, BV510, BD Biosciences), and anti-CD45 (1 µg/100 µl, Percp-cy5.5, BD Biosciences) at RT for 50 min. For NK-cell staining, cells were incubated with anti-NK49b (1 µg/100 µl, FITC, BD Biosciences), anti-CD3, and anti-CD45. Stained cells were collected with BD Verse (BD Bioscience) and analyzed with FlowJo software (Tree Star, Ashland, OR, USA).

### Mouse brain sections

3 w after the intravenous injection of AAV-PHP.eB, the mouse was anesthetized and transcardially perfused with 10 mL of 0.9% NaCl, followed with 20 mL of cold 4% paraformaldehyde (PFA) in PBS for 5 min. The mouse brain was taken out, post fixed in 4% PFA at 4 °C for 4 h, and immersed in 20% and 30% sucrose in PBS at 4 °C for 3 d. Then, the brain of the mouse was sectioned in 25 µm thickness in a cryostat (Leica Microsystems, Wetzlar, Germany). The sections were mounted on gel-coated glass slides and dried at 37 °C for 30 min, which were ready for use or stored at -80 °C.

To monitor the expression of the *Scarlet* or *Egfp* gene in the mouse brain, the slides were coverslipped with an anti-fade mounting medium with 10 µg/ml Hoechst dye. Images were taken under a confocal Laser Scanning Microscope (FV3000, Olympus, Tokyo, Japan) with the same settings. Cells were counted on 40× fluorescence images in various CNS areas. There were three or four mice in each group. The NIH ImageJ (1.52a) program was used to analyze the fluorescence intensity in the various regions of the CNS.

### Immunofluorescent staining

To determine what kinds of cells in the brain AAV-PHP.eB could transduce, sections were stained with rabbit anti-NeuN (GB11138, Servicebio, Wuhan, China), rabbit anti-GFAP (ab7260, abcam, Cambridge, MA, USA), goat anti-Doublecortin (Santa Cruz Biotechnology, Dallas, Texas, USA), and goat anti-CD31 (AF3628, R&D Systems, Minneapolis, MN, USA).

The preparation of sections for immunofluorescent staining was as follows: sections were 1) rinsed in PBS at RT for 5 min; 2) blocked in 5% bovine serum albumin (BSA) in PBS at RT for 1 h; 3) incubated with primary Abs (diluted in 5% BSA) at RT overnight, including the rabbit anti-NeuN Ab (1:100), rabbit anti-Glial fibrillary acidic protein (GFAP) Ab (1:100), goat anti-doublecortin (DCX) Ab (1:100), and goat anti-CD31 Ab (1:100); 4) washed in PBS, 10 min × 3 times; and, 5) incubated in secondary Abs (diluted in 5% BSA) at RT for 1 h. For primary Abs from rabbit, Alexa Fluor 488-labeled goat anti-rabbit IgG (1:100) were used. Alexa Fluor 488-labeled Donkey anti-goat IgG (1:100) was used for goat primary Ab; 6) washed in PBS, 10 min × 3 times; and, 7) coverslipped using anti-fade mounting medium with or without 10 µg/ml Hoechst dye.

### Stereotactic microinjection of AAV-PHP.eB into mouse brains

AAV-PHP.eB was first diluted in PBS to a final concentration of 5 × 10^12^ v.g./mL. Then, over 10 min, 1.5 µl of the virus was stereotactically microinjected into the dentate gyrus of the hippocampus, positioned 2.3 mm posterior to the bregma, 1.4 mm from the midline, and 2.1 mm under the dura, in wild-type C57BL/6 and BALB/c mice as well as *Apoe^-/-^* and *Ldlr^-/-^* C57BL/6 mice.

To evaluate the cell transduction in the dentate gyrus, mice were sacrificed at 3 w post-microinjection, and brain sections were prepared as described above.

### Statistical analysis

The data in this study were collected. One-way analysis of variance (ANOVA), followed by Tukey and Games-Howell post-hoc tests, in the Statistical Package for Social Sciences 17.0 (SPSS, Chicago, IL, USA) were used for data analyses. Student's t-test with two tails was also used. Statistical significance was defined at *p* ≤ 0.05. The data were shown as the means ± standard errors of the means.

## Results

### ApoE and LDLR are required for intravenous AAV-PHP.eB to penetrate the BBB in the C57BL/6 mouse strain

**Figure 1:**
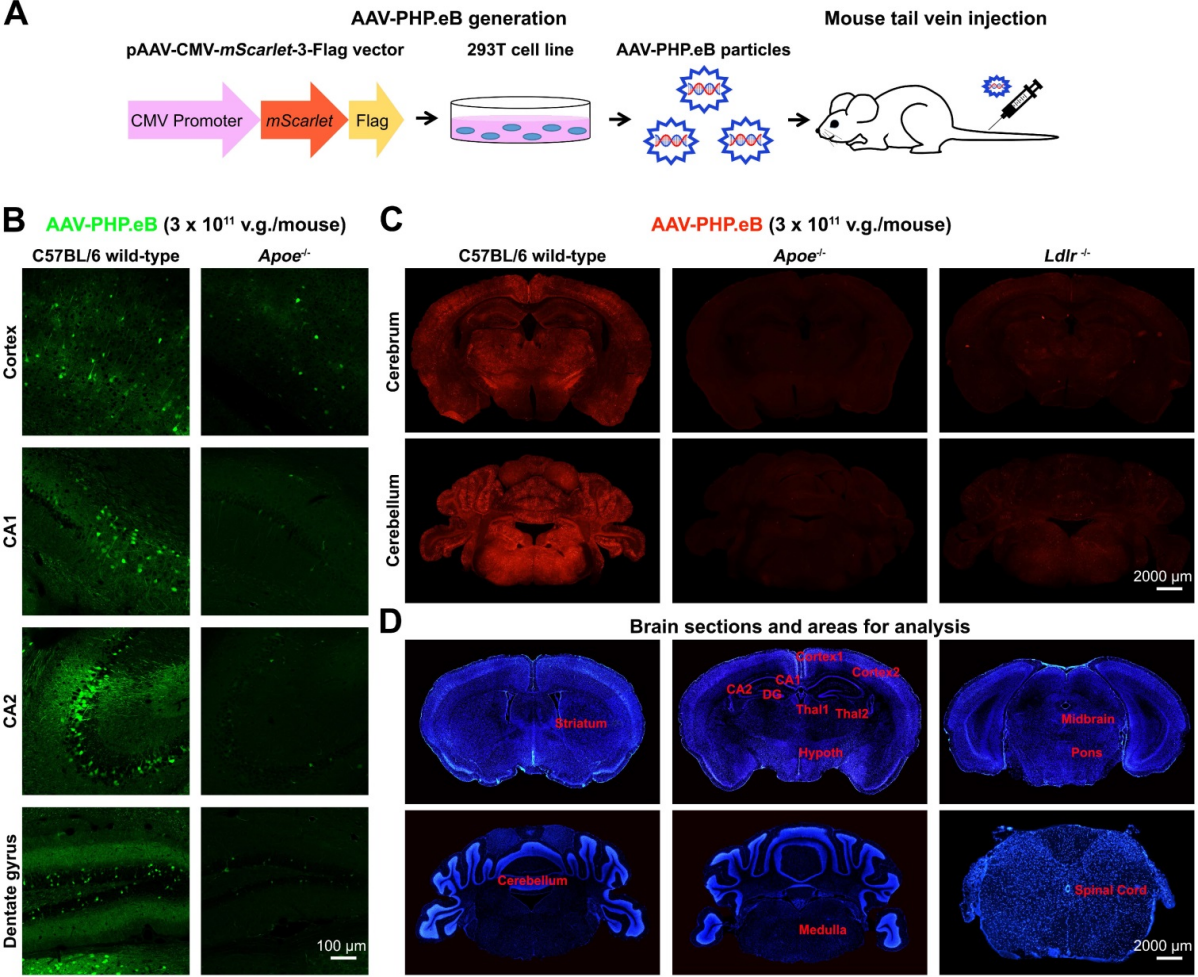
** AAV-PHP.eB preparation and transduction to the mouse central nervous system following intravenous delivery. (A)** Schematic showing the production of AAV-PHP.eB particles carrying the bright red fluorescent protein gene, *mScarlet*, and their administration via the tail vein. **(B)** Transduced cells in the cortex, CA1, CA2 and dentate gyrus 3 weeks after the intravenous administration of AAV-PHP.eB encoding enhanced green fluorescent protein into wild-type and *Apoe* knockout (*Apoe^-/-^*) C57BL/6 mice. **(C)** AAV-PHP.eB-mediated expression of mScarlet in the central nervous systems of wild-type, *Apoe^-/-^*, and *Ldlr* knockout (*Ldlr^-/-^*) C57BL/6 mice, 3 weeks after intravenous injection. **(D)** Brain regions analysed in Fig. S1, including the striatum, cortex1 (primary and secondary motor areas), cortex2 (primary somatosensory area), CA1, CA2, dentate gyrus (DG), thalamus1 (Thal1), thalamus2 (Thal2), hypothalamus (Hypoth), midbrain, pons, medulla, cerebellum and spinal cord. The blue fluorescence indicates Hoechst nuclear staining.

To explore whether ApoE and LDLR modulate the BBB penetration by AAV-PHP.eB, we injected AAV-PHP.eB carrying either the *Egfp* or the *mScarlet* gene intravenously into C57BL/6 mice (Figure 1A). 3 w after the intravenous administration, we observed the strong AAV-PHP.eB transduction throughout the CNS of wild-type C57BL/6 mice, as indicated by fluorescence intensity. In contrast, much less transduction was observed in *Apoe^-/-^* or *Ldlr^-/-^* C57BL/6 mice (Figure 1B-C). Quantification over multiple CNS regions from striatum to spinal cord (Figure 1D) identified significantly lower transduction efficiency in a number of CNS areas of *Apoe^-/-^* and *Ldlr^-/-^* mice compared to the same areas in wild-type C57BL/6 mice (Figure S1A-B). These data indicate that both ApoE and LDLR mediate the intravenous AAV-PHP.eB CNS transduction in the C57BL/6 genetic background.

We next sought to determine whether the *Apoe* or *Ldlr* mutation's effect on the AAV-PHP.eB transduction was specific to the BBB or generic to all the tissues. We examined the AAV-PHP.eB transduction in mouse livers and brains, and found that it was able to transduce both the liver and the brain cells in wild-type C57BL/6 mice, whereas it transduced only the liver cells in *Apoe^-/-^* and *Ldlr^-/-^* mice (Figure S2A). These results suggest that LDLR and ApoE are essential for AAV-PHP.eB to cross the BBB but not to enter the cells of the liver, an organ outside the CNS. In contrast, intravenously administered AAV-9 was unable to transduce to the brains of either wild-type, *Apoe^-/-^*, or *Ldlr^-/-^* C57BL/6 mice, although it transduced their liver cells (Figure S2A). These data suggest that unlike AAV-PHP.eB, AAV-9 cannot recruit ApoE and LDLR to cross the BBB or that other factors such as host immunity impede its barrier penetration. To exclude the possibility that AAV-PHP.eB impacted the BBB permeability, we administered Evans blue or normal saline intravenously in wild-type C57BL/6 mice injected with AAV-PHP.eB. There was no dramatic increase in the Evans blue signal in the brain post-Evan blue injection, as compared to that after normal saline injection (Figure S2B). It indicates that AAV-PHP.eB does not cause the BBB breakdown and that the AAV-PHP.eB CNS transduction isn't the result of its leakage through the BBB in wild-type C57BL/6 mice.

To confirm the role of ApoE in the AAV-PHP.eB CNS transduction, we investigated whether providing exogenous ApoE can rescue the CNS transduction of AAV-PHP.eB in *Apoe^-/-^* mice. Plasma was collected from wild-type C57BL/6 or BALB/c mice, both of which express ApoE, or from *Apoe^-/-^* mice. It was then mixed with AAV-PHP.eB prior to intravenous administration to *Apoe^-/-^* mice. Interestingly, more transduction to the CNS was observed in *Apoe^-/-^* mice receiving the wild-type C57BL/6 or BALB/c mouse plasma than those receiving the *Apoe^-/-^* mouse plasma (Figure S3A‑C). The difference was most significant in the cerebellum, the thalamus, and the medulla (Figure S3C). These findings confirm the essential role of ApoE in the transduction of intravenous AAV-PHP.eB to the CNS. Moreover, they indicate that ApoE from both C57BL/6 and BALB/c mice can mediate this transduction.

### Most AAV-PHP.eB-transduced cells are NeuN-labeled neurons

**Figure 2:**
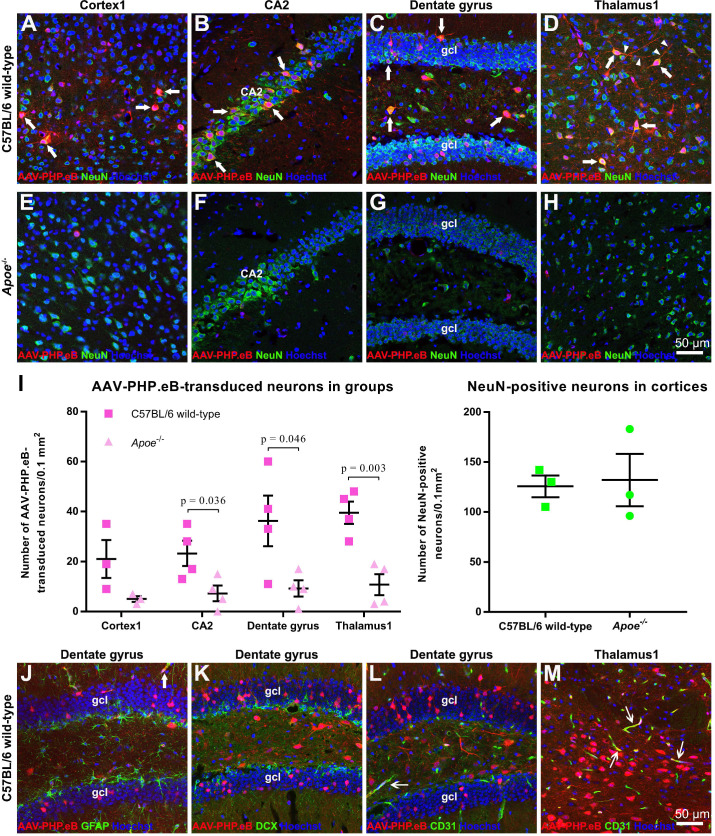
** Identification of AAV-PHP.eB-transduced brain cells via immunofluorescence staining. (A-H)** Co-localization (arrows) of the AAV-PHP.eB-transduced cells (red) and NeuN-positive cells (green) in the indicated tissues in wild-type and *Apoe^-/-^* C57BL/6 mice, 3 weeks after the intravenous AAV-PHP.eB administration. The blue fluorescence indicates Hoechst nuclear staining. gcl: granule cell layer. **(I)** Density of AAV-PHP.eB-transduced neurons (left, n = 4 for each group) and the NeuN-positive neurons (right, n = 3 for each group) in the indicated brain areas and mice; p values were determined by two-tailed Student's t-test. Data are mean ± s.e.m. **(J-M)** Immunofluorescent staining (green) of glial fibrillary acidic protein in the dentate gyrus (**J**); doublecortin (DCX) in the dentate gyrus (**K**); and CD31 in the dentate gyrus and thalamus1 (**L** and **M**), 3 weeks after intravenous administration of AAV-PHP.eB (red). The blue fluorescence indicates Hoechst nuclear staining. Arrows indicate co-localization. gcl: granule cell layer.

We found that AAV-PHP.eB transduced cells were primarily NeuN-positive neurons (Figure 2A‑H). Consistent with this notion, statistical analyses identified significantly more double-labelled cells in the CA2, dentate gyrus, and thalamus of C57BL/6 mice than those of *Apoe^-/-^* mice (Figure 2I). To reject the possibility that the smaller number of double-labelled cells in *Apoe^-/-^* mice was due to a reduced neuron population, we compared the number of NeuN-positive neurons in wild-type and *Apoe^-/-^* C57BL/6 mice. No significant difference in the neuron population was found between two groups (Figure 2I), suggesting that the decrease in the number of AAV-PHP.eB transduced neurons in *Apoe^-/-^* mice is not due to low neuron population.

Our data also showed that some AAV-PHP.eB-transduced cells were CD31-labelled blood vessel endothelial cells (thin arrows), while glial fibrillary acidic protein-stained astrocyte foot networks encircled a transduced blood vessel-like structure (Figure 2J‑M, large arrow). These findings indicate that AAV-PHP.eB can also transduce the BBB while crossing it.

### BALB/c mice are less susceptible than C57BL/6 mice to the AAV-PHP.eB transduction to the CNS

**Figure 3:**
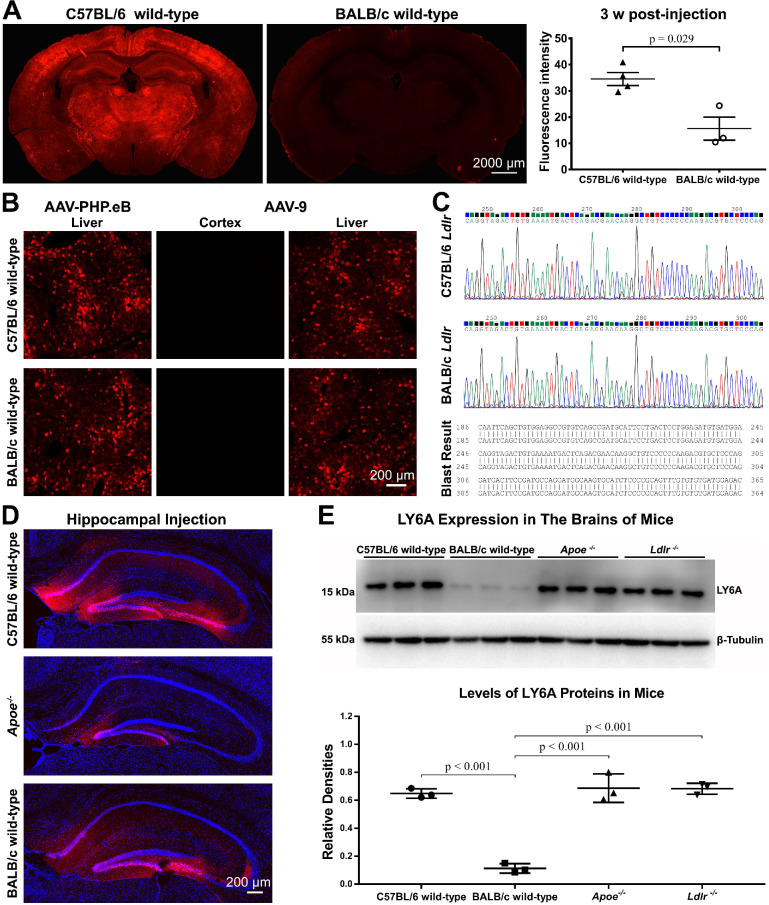
** Exploring factors that modulate the transduction of AAV-PHP.eB to brain cells—mouse strain, AAV serotype, cell surface receptors, injection site, and LY6A level. (A)** Representative images and quantification of the AAV-PHP.eB transduction in the brains of C57BL/6 (n = 4) and BALB/c mice (n = 3), 3 weeks (w) after intravenous injection; the p value was determined by two-tailed Student's t-test. Data are mean ± s.e.m. **(B)** Transduction of intravenous AAV-PHP.eB and AAV-9 to the brain and liver in indicated mouse strains. **(C)** Comparison of C57BL/6 and BALB/c* Ldlr* gene sequences. **(D)** Local transduction of AAV-PHP.eB post-stereotactic microinjection into the hippocampi of the indicated mice (n = 2 for each group). The blue fluorescence indicates Hoechst nuclear staining. **(E)** LY6A levels detected by western blots and its quantification in the indicated mice (n = 3 for each group). The *p* values were determined by Tukey post-hoc tests. Data are mean ± s.e.m.

C57BL/6 and BALB/c are both commonly used laboratory mouse strains. Nevertheless, much less CNS transduction of systemically delivered AAV-PHP.eB was observed in BALB/c mice than that in C57BL/6 mice, and statistical analysis showed this difference to be significant (Figure 3A). In contrast, AAV-PHP.eB was able to transduce both C57BL/6 and BALB/c mouse liver cells (Figure 3B). These data made us wonder whether the BBB of BALB/c mice prevented the transduction of AAV-PHP.eB to the CNS and if it is because of a dysfunctional LDLR.

Meanwhile, intravenous AAV-9 failed to transduce to the brain in either C57BL/6 or BALB/c mice in spite of its ability to transduce the liver cells in these two mouse strains (Figure 3B). These results suggest a lower BBB-penetrating efficiency of AAV-9 compared to that of AAV-PHP.eB.

### BALB/c LDLR can enhance the AAV-PHP.eB CNS transduction in *Ldlr^-/-^* C57BL/6 mice

Since we had shown that ApoE from BALB/c mice could rescue the AAV-PHP.eB CNS transduction in *Apoe^-/-^* mice, we evaluated whether LDLR on the BBB of BALB/c mice accounted for their resistance to the CNS transduction by intravenous AAV-PHP.eB.

Three separate experiments were performed to investigate this. First, we sequenced *Ldlr* genes in BALB/c and C57BL/6 mice. BLAST analysis revealed no differences between these two sequences (Figure 3C). Second, we assessed whether BALB/c mouse brain cells resisted the AAV-PHP.eB transduction. We found that following local stereotactic microinjection of AAV-PHP.eB into the mouse dentate gyrus, fluorescence was observed broadly in the dentate gyrus of C57BL/6 mice and BALB/c mice (Figure 3D). These results indicate that AAV-PHP.eB is capable of transducing the neurons in both C57BL/6 and BALB/c mice. Surprisingly, injection of AAV-PHP.eB in the dentate gyrus led to less transduction in *Apoe^-/-^* mice (Figure 3D), though not in *Ldlr^-/-^* mice (Figure S4). These results indicate that ApoE and LDLR in the mouse CNS may have different roles in the local AAV-PHP.eB transduction versus BBB crossing.

**Figure 4:**
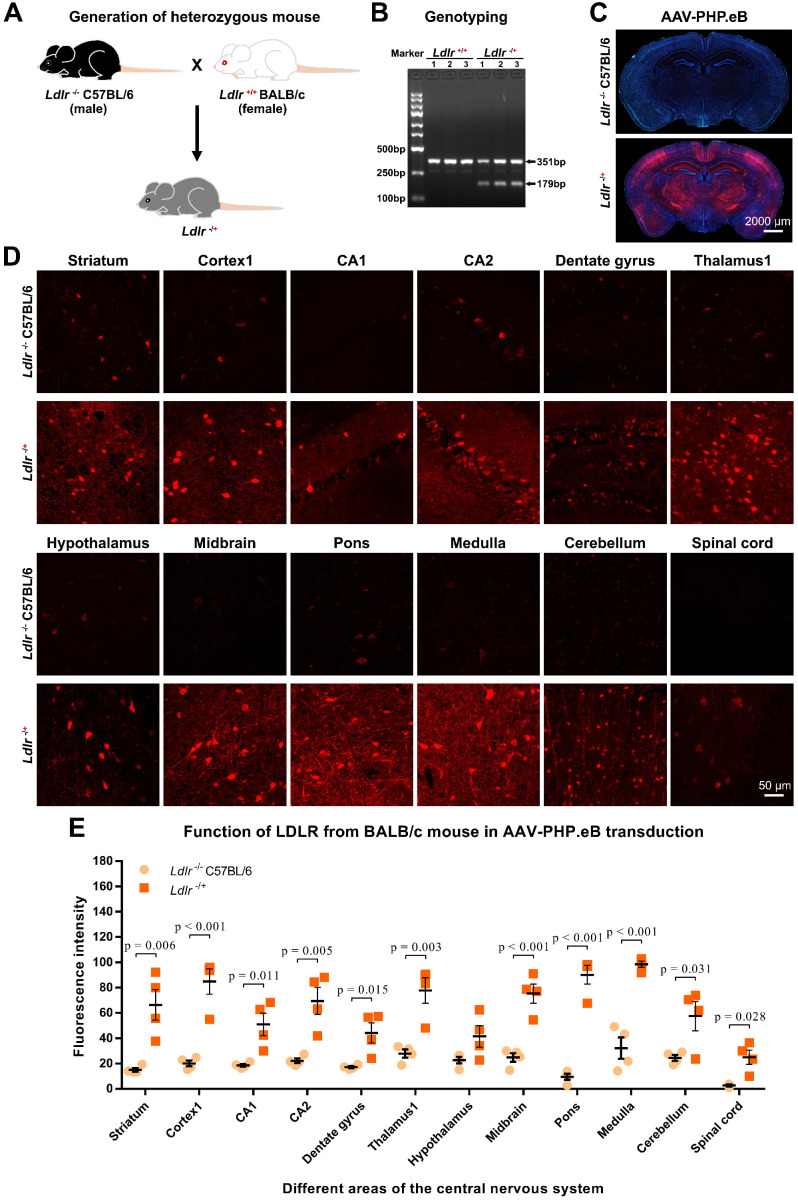
** Effect of the BALB/c-derived LDLR on the AAV-PHP.eB transduction. (A)** Schematic showing the generation of heterozygous knockout (*Ldlr^-/+^*) mouse hybrids expressing the BALB/c *Ldlr* gene. **(B)** Genotyping of *Ldlr^-/+^*mouse hybrids. **(C, D)** Low-magnification (**C**) and high-magnification (**D**) images showing transduction of AAV-PHP.eB expressing *mScarlet* in the indicated brain regions of *Ldlr^-/-^* C57BL/6 and *Ldlr^-/+^* mice 3 weeks after intravenous administration. The blue fluorescence indicates Hoechst nuclear staining. **(E)** Fluorescence intensity in the indicated brain regions of *Ldlr^-/-^* and the *Ldlr^-/+^*mice, showing the mean ± s.e.m. (n = 4 for each group); p values were determined by two-tailed Student's t-test.

Third, we generated heterozygous *Ldlr* knockout (*Ldlr^-/+^*) mice carrying the *ldlr* gene from wild-type BALB/c (Figure 4A-B). Intriguingly, intravenous administration of AAV-PHP.eB led to dramatically elevated transduction throughout the brains of *Ldlr^-/+^* mice compared to that observed in *Ldlr^-/-^* mice (Figure 4C-E). These data suggest that BALB/c LDLR is able to mediate the penetration of the BBB by AAV-PHP.eB.

### LY6A protein level is not affected in the brains of either *Apoe^-/-^* or *Ldlr^-/-^* C57BL/6 mice

Since LY6A has been reported to mediate BBB penetration by AAV-PHP.B in C57BL/6 and BALB/c mice 17-19, we investigated whether the LY6A protein level was changed in the brains of *Apoe^-/-^* or *Ldlr^-/-^* mice. In our western blots, the anti-LY6A monoclonal Ab recognized one protein band with a molecular weight of around 15 kDa. Statistical analyses showed that the LY6A protein level was not affected in either *Apoe^-/-^* or *Ldlr^-/-^* mice compared to that in wild-type C57BL/6 mice, while it was significantly lower in wild-type BALB/c mice than those in mutant or wild-type C57BL/6 mice (Figure 3E). These results are in accordance with a previous report showing a dramatic decrease in the LY6A level in BALB/c mice versus that in C57BL/6 mice 18. Moreover, our data suggest that the absence of ApoE or LDLR doesn't affect the LY6A level in the mouse brains.

### NK-cell depletion improves the AAV-PHP.eB transduction in wild-type BALB/c mice

As we showed above, little CNS transduction of intravenous AAV-PHP.eB was observed in wild-type BALB/c mice, even though ApoE and LDLR from wild-type BALB/c mice can rescue the transduction in *ApoE^-/-^* and *ldlr^-/-^* mice. These findings indicate that the ApoE-LDLR pathway on the BBB doesn't account for the failure of the CNS transduction by AAV-PHP.eB in BALB/c mice. We therefore hypothesized that the AAV-PHP.eB transduction to the CNS might be hampered by BALB/c immune system.

**Figure 5:**
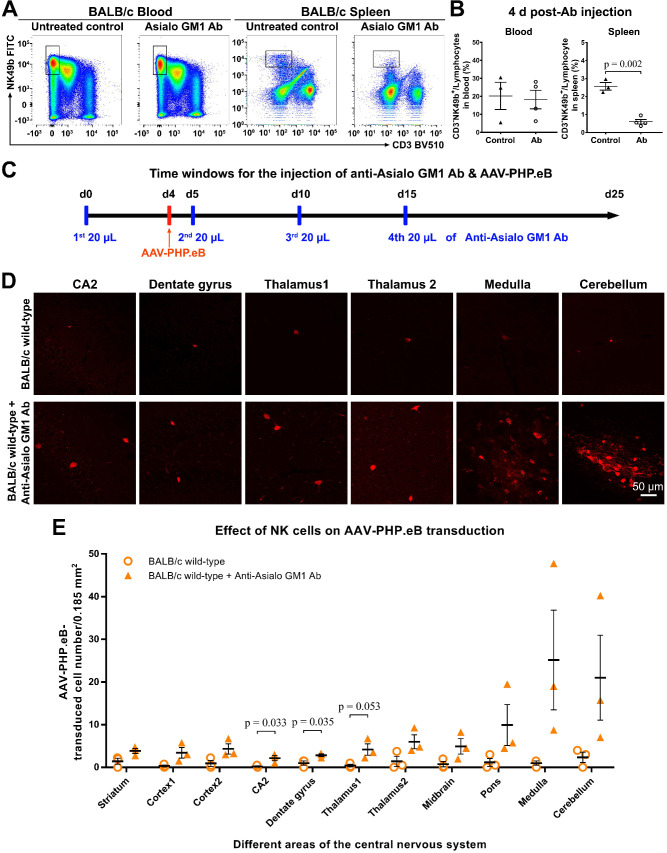
** Influence of natural killer (NK)-cell depletion on AAV-PHP.eB transduction to the CNS in BALB/c mice. (A)** Flow cytometry analyses of NK cells in the blood and spleen of BALB/c mice 4 days (d) after the intraperitoneal administration of anti-Asialo GM1 antibody (n = 4). The untreated BALB/c mice served as controls (n = 3). **(B)** Relative NK cell populations in the blood and spleen of untreated BALB/c mice and antibody-treated BALB/c mice; the p value was determined by two-tailed Student's t-test. **(C)** A schematic showing the timing of anti-Asialo GM1 antibody administration and AAV-PHP.eB injection in BALB/c mice. **(D)** Representative images of AAV-PHP.eB transduction in the indicated mouse brain regions, 3 weeks after intravenous AAV-PHP.eB administration. The untreated BALB/c mice served as controls. **(E)** The densities of AAV-PHP.eB-transduced cells in the indicated brain areas of untreated BALB/c mice and anti-Asialo GM1 antibody-treated BALB/c mice; n = 3 for each group; p values were determined by two-tailed Student's t-test. Data are mean ± s.e.m.

To test this hypothesis, we first depleted the NK cells in BALB/c mice by injecting anti-Asialo GM1 Ab intraperitoneally. Our flow cytometry data showed that compared with untreated mice, the Ab-treated BALB/c mice had significantly fewer NK cells in the spleen, but not in the blood, 4 d after the Ab injection (Figure 5A, B); this was consistent with a previous report by Nishikado et al. 23. To maintain the NK-cell depletion for 20 d, three additional Ab injections were made (Figure 5C). 3 w after the intravenous administration of AAV-PHP.eB, transduction was significantly augmented in the CA2, dentate gyrus, and thalamus1 regions of the brains of BALB/c mice whose NK cells were depleted compared to control mice (Figure 5C-E). These data indicate that NK cells limit the CNS transduction of intravenous AAV-PHP.eB in BALB/c mice.

### Blood B cells, but not T cells, prevent the AAV-PHP.eB transduction to the CNS in wild-type BALB/c mice

**Figure 6:**
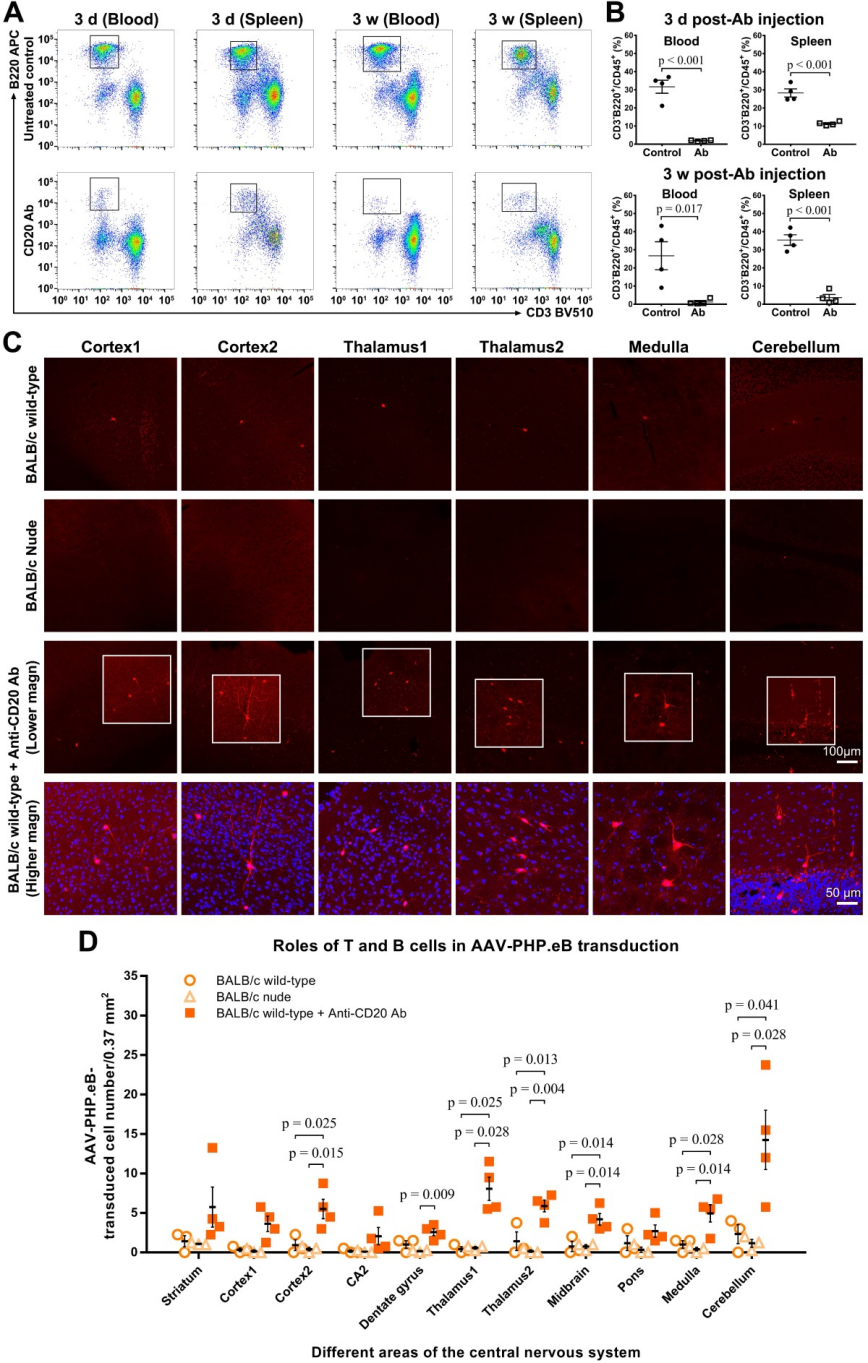
** The AAV-PHP.eB transduction in either B cell- or T cell-deficient BALB/c mice. (A)** Flow cytometry analyses of the B cell population in the blood and spleen of BALB/c mice 3 days (d) and 3 weeks (w) after a single intravenous dose of the anti-CD20 antibody, as compared with untreated wild-type BALB/c mice (Untreated BALB/c control). **(B)** Relative levels of B cells in the blood and spleen of untreated BALB/c mice (control) and antibody-treated BALB/c mice, determined by flow cytometry (n = 4 for each group); p values were determined by two-tailed Student's t-test. **(C)** Representative images of transduced cells in the indicated brain regions 3 w after the intravenous administration of AAV-PHP.eB to untreated BALB/c mice, BALB/c nude (T cell-deficient) mice, and antibody-treated (B cell-deficient) BALB/c mice. The blue fluorescence indicates Hoechst nuclear staining. magn: magnification. **(D)** Quantification of AAV-PHP.eB-transduced cells in the indicated brain areas of the indicated mice 3 w after the intravenous AAV-PHP.eB administration; n = 3 for untreated BALB/c mice and for BALB/c nude mice; n = 4 for antibody-treated BALB/c mice; p values were determined by Games-Howell post-hoc test (thalamus1) or Tukey post-hoc test (all other regions). Data are mean ± s.e.m.

To evaluate whether B cells impacted the AAV-PHP.eB transduction to the CNS in BALB/c mice, we intravenously injected a single dose of anti-CD20 murine IgG2a monoclonal Ab to ablate B cells in BALB/c mice prior to the systemic administration of AAV-PHP.eB (Figure 6). At either 3 d or 3 w after Ab injection, dramatic decreases in B cell numbers were detected by flow cytometry in both blood and spleen of Ab-treated mice compared to untreated wild-type BALB/c mice (Figure 6A-B). These data show that a single dose of the anti-CD20 Ab is able to deplete B cells from the BALB/c mouse significantly for at least 3 weeks.

We found that 3 w after intravenous administration of AAV-PHP.eB, transduction to the brain was significantly higher in B cell-depleted BALB/c mice than that in untreated BALB/c mice (Figure 6C-D). Likewise, C.B-17 SCID mice, which are similar to BALB/c but lack both T and B cells due to *Prkdc^scid^* mutation, showed a significant increase in the AAV-PHP.eB brain transduction after intravenous injection compared with BALB/c mice (Figure S5). These data indicate that B cells downregulate the AAV-PHP.eB CNS transduction in BALB/c mice.

To examine the role of T cells in the CNS transduction of intravenous AAV-PHP.eB, we used BALB/c nude mice, which lack a thymus and are thus deficient in mature T cells. No obvious difference in the CNS transduction of systemically delivered AAV-PHP.eB was observed between BALB/c and BALB/c nude mice (Figure 6C-D). These findings suggest that T cells do not interfere with the brain transduction of intravenous AAV-PHP.eB in BALB/c mice.

Since B cells play a negative role in the AAV-PHP.eB CNS transduction in BALB/c mice, we compared the proportion of B cells in *Ldlr^-/+^* hybrids carrying the mixed background of BALB/c and C57BL/6 mice, with that in *Ldlr^-/-^*C57BL/6 mice. The result showed there was no significant difference in B-cell proportion between *Ldlr^-/+^*and *Ldlr^-/-^*mice (Figure S6). This finding rules out the possibility that a change in B-cell abundance leads to the increased CNS transduction of AAV-PHP.eB in *Ldlr^-/+^*mice.

## Discussion

In this study, we have systematically addressed the unknown mechanisms underlying the BBB penetration by AAV-PHP.eB, an efficient gene delivery system, and why such barrier penetration occurs in C57BL/6 but not in BALB/c mice. We found that the intravenously injected AAV-PHP.eB transduction to the CNS, a proxy for BBB penetration, depends on the ApoE-LDLR pathway in both C57BL/6 and BALB/c mice and is limited by NK and B cells, but not T cells, in BALB/c mice.

### ApoE and LDLR mediate AAV-PHP.eB crossing through the BBB

Both *in vitro* and *in vivo* studies have shown that the efficiency with which different AAVs transduce to the CNS, reflecting their ability to penetrate the BBB, varies from serotype to serotype. For instance, AAV-9 crosses the endothelium of the BBB more effectively than AAV-2 does 24. Similarly, the CNS transduction of AAV-PHP.eB is more efficient than that of AAV-PHP.B, whose efficiency is much better than the one of AAV-9 12,15. Although studies indicate that the cellular receptor, LY6A, may be related to the AAV-PHP.eB tropism toward the CNS 17-19, the differences between serotypes have not been elucidated in depth.

Accumulating evidence has demonstrated that virus transduction into cells entails receptor-dependent attachment and subsequent cellular internalization and trafficking 9,25-27. For example, studies have shown that AAV interactions with specific glycoprotein receptors on cell surfaces account for the transduction of the various AAV serotypes, including AAV-1, 2, 5, 8, and 9 25,26. Meanwhile, the cell surface receptor, LDLR, has been reported to contribute to hepatitis C virus entry into hepatocytes 22.

LDLR is known to mediate the cellular uptake of cholesterol-carrying lipoproteins, yet it has also been implicated in the BBB crossing 20,28,29. In particular, the ApoE-LDLR pathway appears to play a dominant role in the delivery of intravenously injected therapeutic peptides/proteins and some nanoparticles to the brain through the BBB 21,30,31. For instance, a study indicates that peptides binding LDLR can facilitate the uptake of intravenous Ab Fc fragments by the mouse brain 21. In addition, PBCA nanoparticles used as intravenous delivery vehicles to target medicines to the brain rely on the ApoE-LDLR pathway-mediated transcytosis on the endothelial cells of the BBB 30. In our current studies, we found that the ApoE-LDLR pathway was also essential to BBB penetration by AAV-PHP.eB, which illustrates the importance of this pathway in the transduction of AAV to the CNS for therapeutic purposes.

In addition, we found that following local injection into the brain, there was less AAV-PHP.eB transduction in the dentate gyrus in *Apoe^-/-^* mice than that in *Ldlr^-/-^* or wild-type C57BL/6 mice. Our data indicate that the ApoE deficiency seemingly affects the local transduction of AAV-PHP.eB to the cells in the brain and that the absence of ApoE has more influence on the local transduction than LDLR deficiency does. These findings may reflect the fact that ApoE can bind to all other the members of the LDLR family, such as LDLR-related protein and ApoE receptor 2, other than LDLR itself in the CNS, whereas ApoB and ApoE are the only extracellular ligands of LDLR 29,32,33.

### NK cells and B cells limit the BBB penetration by AAV-PHP.eB in BALB/c mice

Although C57BL/6 and the BALB/c mice belong to the same species, they have been reported to yield different results in a number of experiments 13,34. Differences were even reported between genders and substrains of C57BL/6 35-37. In our study, we observed a greatly reduced transduction of AAV-PHP.eB to the CNS in BALB/c mice compared to C57BL/6 mice following systemic delivery. However, further findings indicated that the ApoE-LDLR pathway is not responsible for the decreased CNS transduction of intravenously administered AAV-PHP.eB in BALB/c mice.

It has been reported that systemically delivered viral vectors can trigger host immune responses to the viral capsid and the transgene products 38,39.

The immune system acts as a barrier providing the host a defence against pathogens. Increasing evidence shows that NK cells are a crucial defense against viral infection 40-42. Consistent with this notion, we found that depleting BALB/c mice of NK cells increased significantly the transduction of intravenous AAV-PHP.eB to the CNS, indicating that NK cells are one of the factors which attenuate the CNS transduction in BALB/c mice.

The anti-asialo GM1 Ab we used to deplete NK cells has been reported to also decrease basophils counts 23. Nevertheless, due to NK cells' larger population and more dominant role in the response to viruses than the basophils' 42,43, we infer that the elevation in the CNS transduction of AAV-PHP.eB in the mice is mainly a result of NK-cell depletion.

Our findings also implicate B cells in BALB/c's resistance to the CNS transduction by AAV-PHP.eB. Indeed, we observed a dramatic increase in the AAV-PHP.eB brain transduction in B cell-deficient mice, including the B cell-depleted BALB/c mice as well as both B cell and T cell-deficient C.B-17 SCID mice. B cells are mediators of the adaptive immune response. They express unique B-cell receptors that recognize particular antigens, and produce antibodies to neutralize pathogens and present antigens to the T cells, which kill the infected cells and activate other immune cells 44.

Epidemiological studies have identified AAV-neutralizing antibodies in human serum samples 45-47, which indicates a previous AAV infection and the subsequent activation of AAV-specific B cells. The activated B cells can be permanently retained in the host as immune memory cells, which produce more neutralizing antibodies following re-exposure to the relevant antigen 48,49.

Although preexisting AAV-neutralizing antibodies could neutralize intravenous AAV-PHP.eB and impede its CNS transduction, they are probably not the main reason behind AAV-PHP.eB's failure to cross the BBB in BALB/c mice. First, their titer is not expected to drop immediately after B-cell removal. Second, even if memory B cells produce anti-AAV neutralizing antibodies after an intravenous injection of AAV-PHP.eB, this effect takes several weeks, by which time AAV-PHP.eB should already have transduced to the CNS. Thus, a plausible explanation is that the B-cell receptor, an immunoglobulin on B cells, interacts with intravenous AAV-PHP.eB, which then prevents AAV-PHP.eB from binding to ApoE and LDLR for the BBB penetration.

Clinical trials have indicated that AAV triggers an immune response mediated by capsid-specific T cells, including both CD8^+^ and CD4^+^ T cells 49-51. However, our findings showed that T cells might not play a critical role in preventing the transduction of intravenous AAV-PHP.eB to the brain in BALB/c mice. More studies are needed to elucidate the precise role of T cells in AAV-PHP.eB BBB penetration.

Taken together, our findings in immunodeficient mice demonstrate that B cells and NK cells, but not T cells, interfere with the CNS transduction of intravenous AAV-PHP.eB in BALB/c mice.

### The ApoE-LDLR pathway is one of the multiple mechanisms underlying the BBB penetration by AAV-PHP.eB

Recent studies have reported that the cell surface receptor, LY6A, drives the AAV-PHP.eB penetration of the BBB in C57BL/6 mice 17-19 and that the failure of AAV-PHP.eB to cross the BBB in BALB/c mice is due to the absence of LY6A on the BBB in these mice 18. By contrast, here we have shown that AAV-PHP.eB depends on ApoE and its cell surface receptor, LDLR, to cross the BBB in C57BL/6 mice. Our findings that ApoE and LDLR mutations could reduce the BBB penetration by AAV-PHP.eB in spite of normal LY6A levels indicate that the ApoE-LDLR pathway is another important route for AAV-PHP.eB to cross the BBB. In addition, if AAV-PHP.eB relied only LY6A for the BBB penetration, then no CNS transduction of intravenous AAV-PHP.eB should occur in BALB/c mice, which lack LY6A, under any circumstances. In our study, however, we noted that the depletion of either B cells or NK cells significantly enhanced AAV-PHP.eB CNS transduction in BALB/c mice. Taken together, our findings suggest that multiple mechanisms are involved in CNS transduction by the intravenous AAV-PHP.eB in mice.

## Supplementary Material

Supplementary figures.

## Abbreviations

AAV: adeno-associated virus
Ab: antibody
ApoE: apolipoprotein E
BBB: blood-brain barrier
CMV: cytomegalovirus
CNS: central nervous system
EGFP: enhanced green fluorescent protein
gcl: granule cell layer
HCV: hepatitis C virus
KO: knockout
LDLR: low-density lipoprotein receptor
magn: magnification
NK cells: natural killer cells
PCR: polymerase chain reaction
