# Supplementary material for: Apolipoprotein E, low-density lipoprotein receptor, and immune cells control blood-brain barrier penetration by AAV-PHP.eB in mice
Source: Theranostics. 2021 Jan 1;11(3):1177–91. doi: 10.7150/thno.46992 (PMC7738887; doi:10.7150/thno.46992)
Supplement: Supplementary file 1 — Supplementary figures. [file thnov11p1177s1.pdf]

Theranostics

Supplementary Materials for

# Apolipoprotein E, low-density lipoprotein receptor, and immune cells control blood-brain barrier penetration by AAV-PHP.eB in mice

Bao-Shu Xie[1,4#], Xin Wang[2#], Yao-Hua Pan[1#], Gan Jiang[3], Jun-Feng Feng[1], and Yong Lin[1*]

[#] These authors contributed equally to this work.

*** To whom correspondence should be addressed:**

Yong Lin, MD, PhD, Department of Neurological Surgery, Ren Ji Hospital, School of Medicine, Shanghai Jiao Tong University, 160 Pujian Road, Shanghai 200127, P.R. China. Tel: +86 (21) 6838 3982. Email: yonglin1996@hotmail.com

**This PDF file includes:** Figures S1 to S6

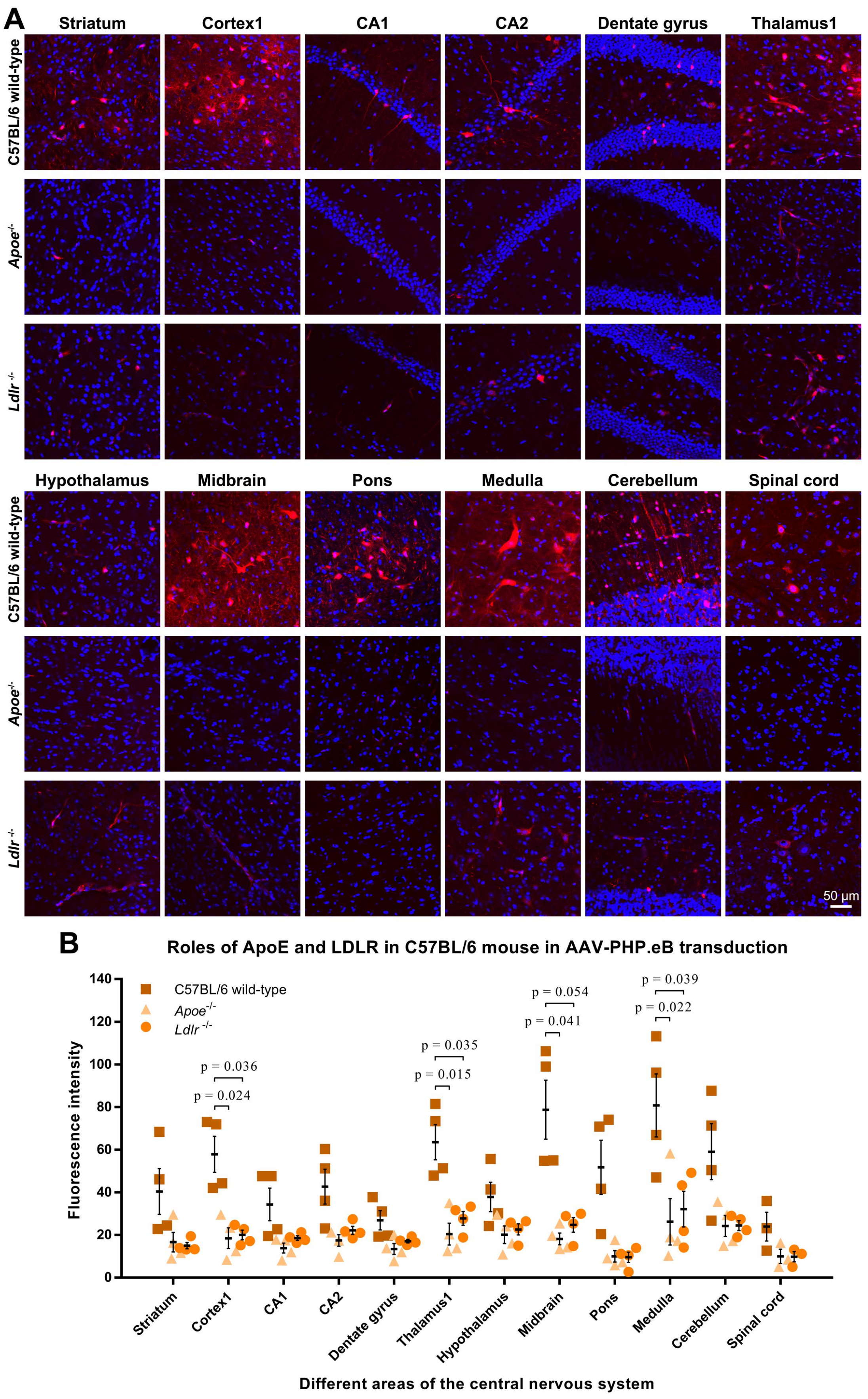
A
Striatum
Cortex1
CA1
CA2
Dentate gyrus
Thalamus1
C57BL/6 wild-type
Apoe-/-
Ldlr -/-
Hypothalamus
Midbrain
Pons
Medulla
Cerebellum
Spinal cord
50 μm
B
Roles of ApoE and LDLR in C57BL/6 mouse in AAV-PHP.eB transduction
C57BL/6 wild-type
Apoe-/-
Ldlr -/-
Fluorescence intensity
0
20
40
60
80
100
120
140
p = 0.036
p = 0.024
p = 0.035
p = 0.015
p = 0.054
p = 0.041
p = 0.039
p = 0.022
Striatum
Cortex1
CA1
CA2
Dentate gyrus
Thalamus1
Hypothalamus
Midbrain
Pons
Medulla
Cerebellum
Spinal cord
Different areas of the central nervous system

**Figure S1 | Transduction of intravenous AAV-PHP.eB into various brain regions of C57BL/6 wild-type, *Apoe*$^{-/-}$ and *Ldlr*$^{-/-}$ mice. (A)** Representative fluorescent images of the indicated tissues in the indicated mice following intravenous administration of AAV-PHP.eB. The blue fluorescence indicates Hoechst nuclear staining. **(B)** Quantification of the red fluorescence intensity in the indicated mice and brain regions 3 weeks after the AAV-PHP.eB administration (n = 4 for each group). For the comparison of C57BL/6 wild-type with either *Apoe*$^{-/-}$ or *Ldlr*$^{-/-}$ mice, p values were determined by Tukey post-hoc test (medulla) or Games-Howell post-hoc test (all other regions). Data are mean ± s.e.m.

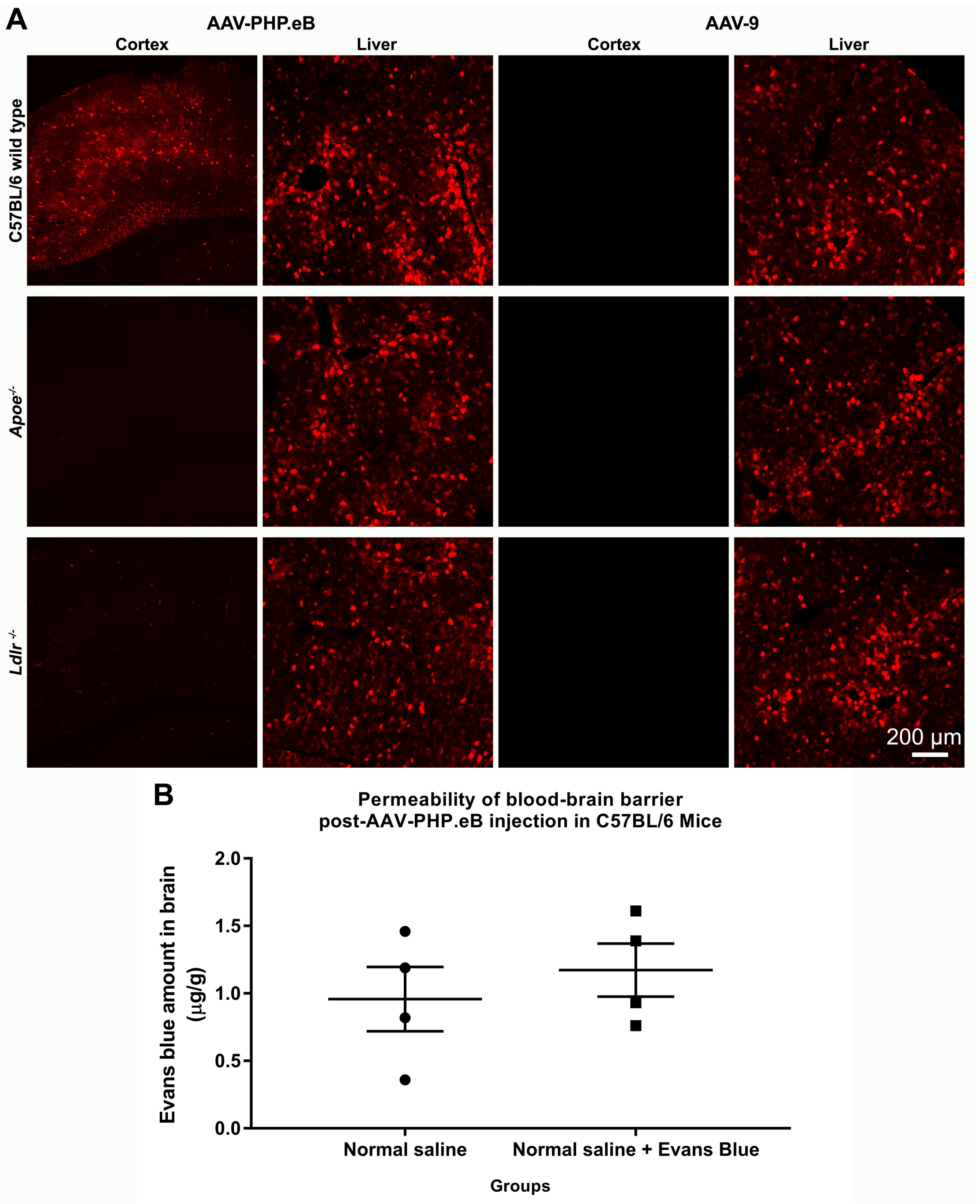


**Figure S2 | Brain and liver transduction of AAV-PHP.eB and AAV-9 and effect of AAV-PHP.eB on the permeability of the blood-brain barrier in mice. (A)** Representative images of the AAV-PHP.eB and AAV-9 transduction in the indicated tissues in the indicated mice. While transducing to both the brain and the liver (red fluorescence) in wild-type C57BL/6 mice, AAV-PHP.eB is able to transduce only the

liver cells in *Apoe*$^{-/-}$ or *Ldlr*$^{-/-}$ mice (n = 3 for each group). In contrast, AAV-9 transduces the liver cells, but fails to transduce brain cells in all three mouse genotypes (n = 3 for each group). **(B)** Evaluation on the permeability of the blood-brain barrier after the AAV-PHP.eB injection. Intravenously injected AAV-PHP.eB does not significantly increase the barrier permeability, measured by Evans Blue infiltration, in C57BL/6 mice (n = 4 for each group).

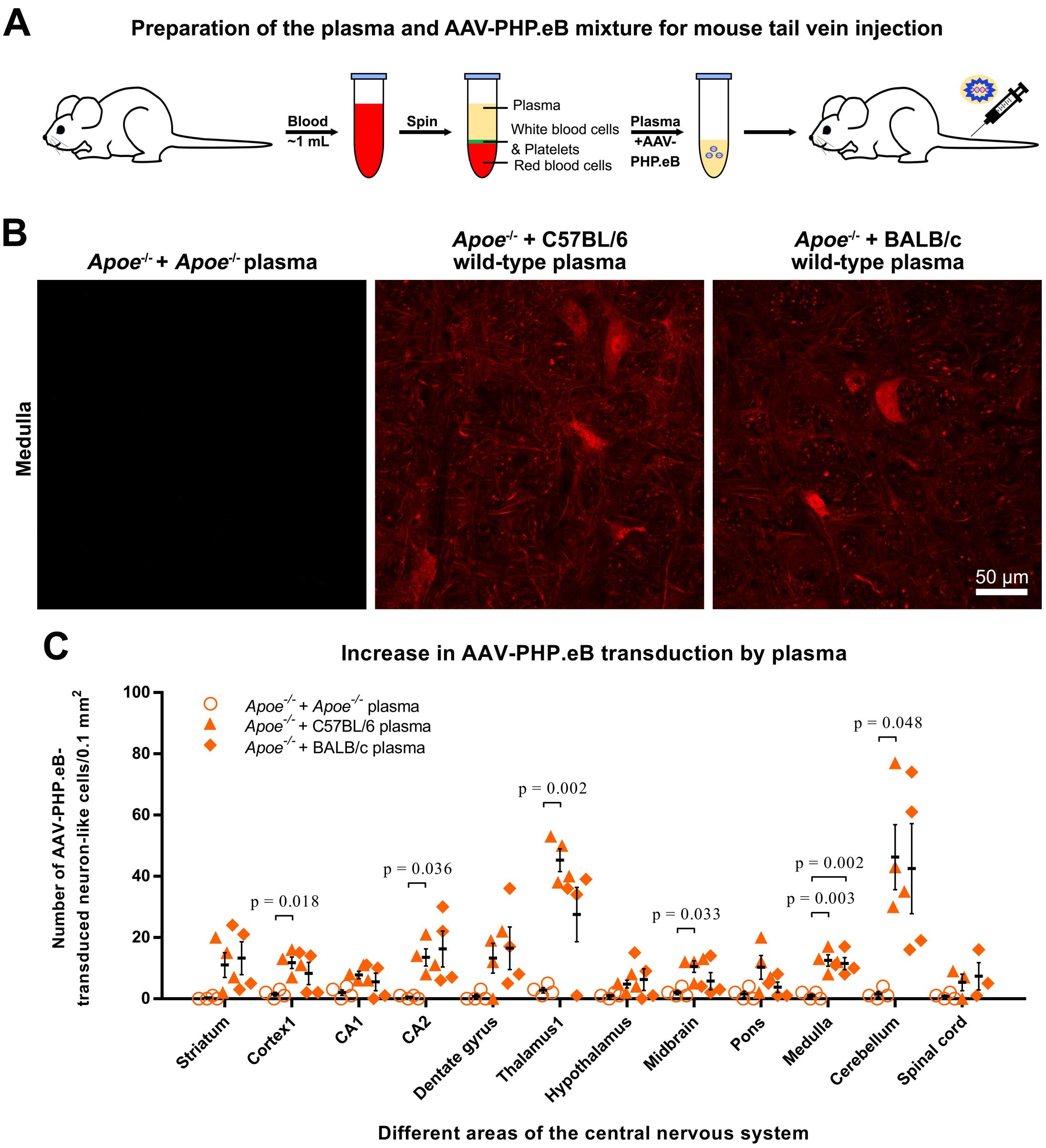


**Figure S3 | Effect of plasma on the central nervous system transduction of intravenous AAV-PHP.eB in *Apoe*$^{-/-}$ mice. (A)** Schematic showing plasma isolation from wild-type mouse blood and preparation of AAV-PHP.eB expressing the *mScarlet* gene mixed with plasma. Thirty minutes after being mixed with ApoE-containing plasma prepared from either C57BL/6 or BALB/c mice, AAV-PHP.eB was administered intravenously to *Apoe*$^{-/-}$ mice. **(B)** Representative images showing transduction in the medulla regions 3 weeks after systemic delivery of AAV-PHP.eB

plus plasma. Images from an *Apoe*$^{-/-}$ mouse which was not treated with plasma are shown as a negative control. **(C)** Quantification of the fluorescence intensity of the indicated brain regions. The p values were determined by one-way ANOVA. The means ± s.e.m are indicated (n = 4 for each group).

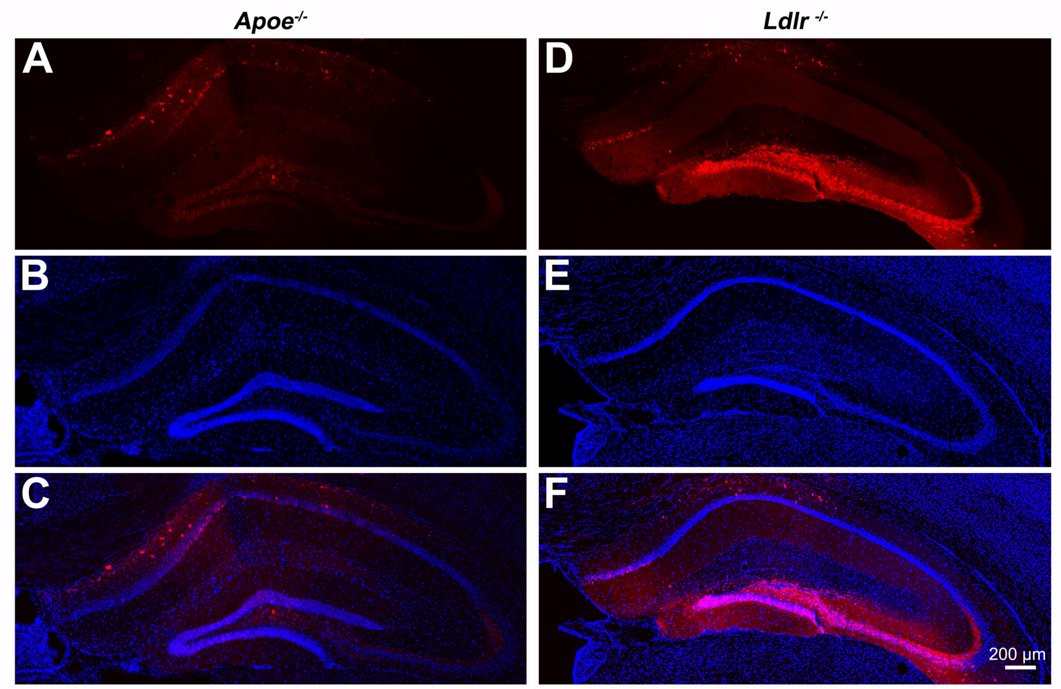


**Figure S4 | Comparison of the local transduction of AAV-PHP.eB in the brains in *Apoe*$^{-/-}$ and *Ldlr* $^{-/-}$ mice.** Representative fluorescent images (red, **A** and **D**) of the hippocampi (**B** and **E**) in *Apoe*$^{-/-}$ (**A-C**) and *Ldlr* $^{-/-}$ **(D-F)** mice following the stereotactic microinjection of AAV-PHP.eB into the dentate gyrus. **A** and **B** are merged in **C**, and **D** and **E** in **F**. The blue fluorescence indicates Hoechst nuclear staining. n = 2 for each group.

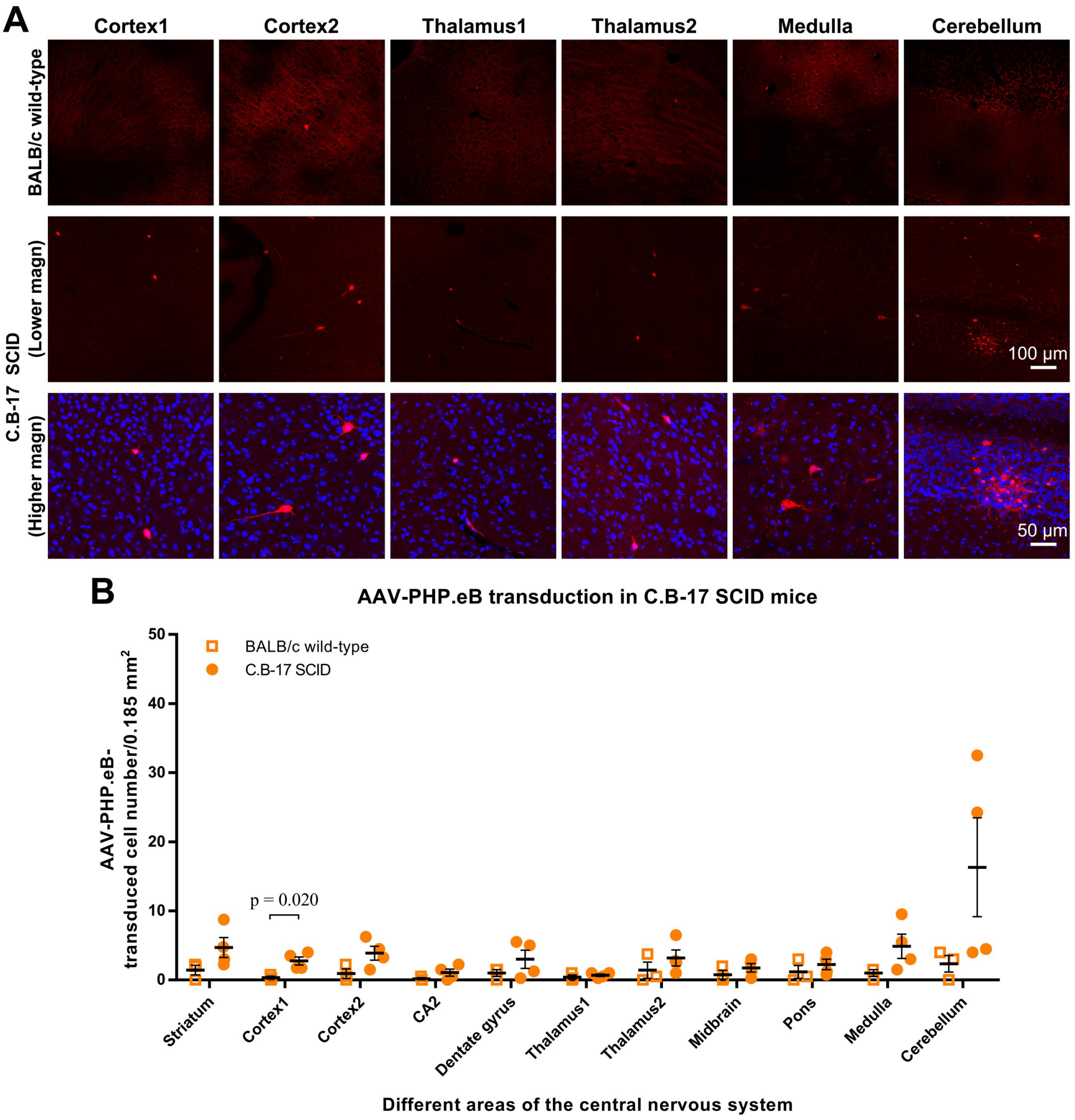


**Figure S5 | Transduction of intravenous AAV-PHP.eB to the brain of in C.B-17 SCID mice lacking both T and B cells. (A)** Representative images of the indicated tissues, 3 weeks after the intravenous AAV-PHP.eB administration to BALB/c wild-type and C.B-17 SCID mice. The blue fluorescence indicates Hoechst nuclear staining. magn: magnification. **(B)** Analyses of the AAV-PHP.eB transduction in the indicated areas of BALB/c (n = 3) and C.B-17 SCID mice (n = 4), 3 weeks after the AAV-PHP.eB injection; the p value was determined by two-tailed Student's t-test. Data are mean ± s.e.m.

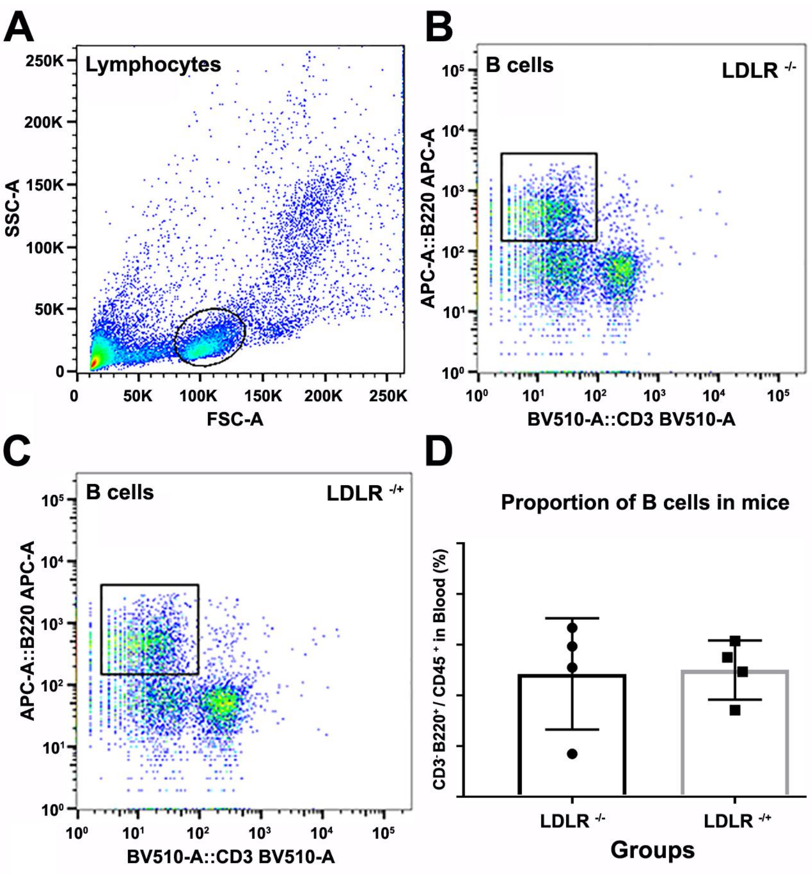


**Figure S6 | Lymphocyte and B cell populations in the spleens of *ldlr*$^{-/-}$ and *ldlr*$^{-/+}$ mice. (A-C)** Flow cytometry analyses of lymphocytes in the spleen (**A**) and of B cells in *ldlr*$^{-/-}$ (**B**) and *ldlr*$^{-/+}$ (**C**) mice. **(D)** Quantification of the ratios of CD3$^-$B220$^+$ B cells to CD45$^+$ lymphocytes. The means ± s.e.m are indicated (n = 4 for each group). The p values > 0.05 (two-tailed Student's t-test).
